# Nicastrin and Notch4 drive endocrine therapy resistance and epithelial to mesenchymal transition in MCF7 breast cancer cells

**DOI:** 10.1186/bcr3675

**Published:** 2014-06-11

**Authors:** Ylenia Lombardo, Monica Faronato, Aleksandra Filipovic, Valentina Vircillo, Luca Magnani, R Charles Coombes

**Affiliations:** 1 Imperial College London, Division of Surgery and Cancer, Department of Oncology, Hammersmith Hospital Campus, Du Cane Road, London, W12 0NN, UK; 2 Department of Pharmacy, Health and Nutritional Sciences, University of Calabria, Via Pietro Bucci, Arcavacada di Rende, CS 87036, Italy

## Abstract

**Introduction:**

Resistance to anti-estrogen therapies is a major cause of disease relapse and mortality in estrogen receptor alpha (ERα)-positive breast cancers. Tamoxifen or estrogen withdrawal increases the dependence of breast cancer cells on Notch signalling. Here, we investigated the contribution of Nicastrin and Notch signalling in endocrine-resistant breast cancer cells.

**Methods:**

We used two models of endocrine therapies resistant (ETR) breast cancer: tamoxifen-resistant (TamR) and long-term estrogen-deprived (LTED) MCF7 cells. We evaluated the migratory and invasive capacity of these cells by Transwell assays. Expression of epithelial to mesenchymal transition (EMT) regulators as well as Notch receptors and targets were evaluated by real-time PCR and western blot analysis. Moreover, we tested *in vitro* anti-Nicastrin monoclonal antibodies (mAbs) and gamma secretase inhibitors (GSIs) as potential EMT reversal therapeutic agents. Finally, we generated stable Nicastrin overexpessing MCF7 cells and evaluated their EMT features and response to tamoxifen.

**Results:**

We found that ETR cells acquired an epithelial to mesenchymal transition (EMT) phenotype and displayed increased levels of Nicastrin and Notch targets. Interestingly, we detected higher level of Notch4 but lower levels of Notch1 and Notch2 suggesting a switch to signalling through different Notch receptors after acquisition of resistance. Anti-Nicastrin monoclonal antibodies and the GSI PF03084014 were effective in blocking the Nicastrin/Notch4 axis and partially inhibiting the EMT process. As a result of this, cell migration and invasion were attenuated and the stem cell-like population was significantly reduced. Genetic silencing of Nicastrin and Notch4 led to equivalent effects. Finally, stable overexpression of Nicastrin was sufficient to make MCF7 unresponsive to tamoxifen by Notch4 activation.

**Conclusions:**

ETR cells express high levels of Nicastrin and Notch4, whose activation ultimately drives invasive behaviour. Anti-Nicastrin mAbs and GSI PF03084014 attenuate expression of EMT molecules reducing cellular invasiveness. Nicastrin overexpression *per se* induces tamoxifen resistance linked to acquisition of EMT phenotype. Our finding suggest that targeting Nicastrin and/or Notch4 warrants further clinical evaluation as valid therapeutic strategies in endocrine-resistant breast cancer.

## Introduction

Resistance to endocrine therapies remains a major challenge in the treatment of estrogen receptor alpha-positive (ERα + ve) breast cancers. Despite an initial responsive phase, many patients will have recurrent endocrine-resistant disease associated with reduced survival [1,2]. Endocrine-resistant tumours frequently display an aggressive phenotype with enhanced metastatic capacity, features that more often typify ERα-negative (ERα-ve) tumours [3]. Consistent with clinical outcomes, quantitative proteomic analysis of acquired tamoxifen resistance cells confirmed signatures associated with an increased migratory capacity [4]. Several studies demonstrated that acquired resistance to drugs that block ER signalling is the consequence of activation of alternative survival signalling via growth factor receptors, such as epidermal growth factor receptor (EGFR) and human epidermal growth factor 2 (HER2), that enable ERα + ve breast cancer cells to escape anti-estrogen actions and contribute to an invasive phenotype [5,6].

Recently, the contribution of Notch pathway in endocrine therapy resistance has been revealed by several studies [7-9]. In particular, it has been demonstrated that Notch signalling is augmented in endocrine therapies resistant (ETR) cells following a global reprogramming of the epigenome. The growth of resistant breast cancer cells can be abrogated by blocking Notch signalling, which is activated in these cells [7]. Moreover, it has previously been shown, in this system, that estradiol inhibits Notch activity by altering Notch receptor cellular localization. Tamoxifen or estrogen withdrawal block this effect and increase the dependence of breast cancer cells on Notch signalling [10]. Hao *et al*., have also demonstrated that Notch activates the transcription of ERα target genes via a nuclear IKKα-dependent pathway [11]. Pharmacological inhibition of Notch activation with gamma-secretase inhibitors (GSIs) in combination with tamoxifen has synergistic effects in ERα + ve breast cancer *in vivo* models [10,11]. Recently, a functional crosstalk between PKCα and Notch4 has been reported in endocrine-resistant breast cancer cells [9].

The Notch signalling pathway is a key regulator of epithelial to mesenchymal transition (EMT) affecting migration and invasion of breast cancer cells [12], and several studies link EMT to the generation of cancer stem cells (CSCs) with mesenchymal and self-renewal features necessary for dissemination and formation of metastasis [13,14].

The Notch signalling pathway has also been implicated in controlling the fate of putative stem cells in the normal human mammary gland [15,16] and in the regulation of CD44^+^CD24- breast CSCs in both ductal carcinoma *in situ* (DCIS) and invasive carcinoma [17-19]. Specifically, it has been found that breast CSCs activity depends on Notch4 receptor signalling and its inhibition can significantly reduce mammosphere formation in primary human DCIS *in vitro* as well as tumour formation *in vivo*[8].

Importantly, we recently highlighted the importance of Nicastrin, a component of the gamma secretase (GS) enzyme, for the expansion of breast CSC population and their invasive features [13]. Nicastrin is highly expressed in breast cancers and confers worse overall survival in ERα-ve tumours [20]. It has also been demonstrated that Nicastrin stable knockdown induces Notch inhibition in basal-like breast cancer cells, sensitising them to anti-proliferative effects of EGFR inhibition [21]. Structurally, Nicastrin is the only GS component with a large extracellular domain that makes this protein a potential target for monoclonal antibody (mAb) therapy. In a recent study, Hayashi *et al*. reported the neutralization of the GS activity by a novel mAb against the extracellular domain of Nicastrin. This antibody abolished the GS activity-dependent growth of cancer cells in a xenograft model [22].

We developed and fully characterized two mAbs against the extracellular domain of Nicastrin that reduce tumour and metastasis formation *in vivo* of triple-negative breast cancer cell lines ([20] and submitted data).

Here, we show the contribution of Notch4 and Nicastrin in the development of endocrine therapies resistance. We present data supporting the efficacy of GSI PF03084014 and anti-Nicastrin mAbs that can reverse and potentially re-sensitize endocrine-resistant breast cancers.

## Methods

### Cell lines, antibodies, mAbs and GSI

MCF7 and tamoxifen-resistant (TAM-R) cells derived from MCF7 as previously described [3] were a kind gift from Dr. Hiscox (Welsh School of Pharmacy, Cardiff University, Wales, UK). Long-term estrogen-deprived cells (LTED) were derived from MCF7 after six-month estrogen deprivation as previously described and were a kind gift from Dr. Hellis. MCF7 cells were maintained in Dulbecco’s modified Eagle’s medium (DMEM) containing 10% fetal calf serum (FCS). TAM-R and LTED were maintained in phenol-red free DMEM containing 10% charcoal stripped fetal calf serum (SFCS). Both media were supplemented with 2 mM L-glutamine, 100 units/mL penicillin, 0.1 mg/mL. 10^-7^ M 4-OH-tamoxifen was added routinely to TAM-R. A mycoplasma test was carried out monthly; cells were used for a limited number of passages. All antibodies were bought from Cell Signaling (Merck KGaA, Darmstadt, Germany) (Notch1 D1E11 3608S, Notch2 5732P, Notch3 5276P, Rac1 2465P, Cdc42 2466P, IQGAP1) with the exception of Notch4, SC-5594, (Santa Cruz Biotechnology, Dallas, Texas, USA) Actin Ab6276, (Abcam, Cambridge, UK) Nicastrin and αTubulin (N1660, clone B512, Sigma-Aldrich, St. Louis, MI, USA) GSI were purchased from Pfizer, Inc (New York, NY, USA) (PF03084014) and Roche (Basel, Switzerland) (RO4929097) and used at 10 μM final concentration. Tamoxifen was purchased from Sigma-Aldrich (H7904). For the generation of Nicastrin stably overexpressing MCF7 cell line, the cDNA encoding full-length human Nicastrin was subcloned into a retroviral expression vector pMXs-puro and the construct was kindly provided by Prof. Gopal Thinakaran, (University of Chicago, USA). The pMXs-puro-nicastrin retroviral vector was used to infect MCF7 cells. Control cells were generated by retroviral transduction of the empty vector pMXs-puro (Cambridge Bioscience, Cambridge, UK). Stable, polyclonal cell populations were established after puromycin selection (0.5 μg/ml).

### Anti-nicastrin monoclonal antibodies

mAbs against Nicastrin extracellular domain were produced by genetic immunization of rats using DNA fragment corresponding to Nicastrin amino acids 34-669 aa (Genovac, Freiburg, Germany). Antibody clones mAb1 and mAb2 were purified and used in subsequent experiments. Rat immunoglobulin G (IgG) (Ab36371) was used as control. In all experiments where antibodies were used, cells were pre-treated with 50 μM (mAbs or control) for 30 minutes at room temperature on a tube rotator before seeding.

### FACS analysis using fluorescein isothiocyanate (FITC)-conjugated antibodies

TAM-R cells were cultured to 70 to 80% confluence and detached from the cell culture flasks using EDTA. Cell pellets were obtained and washed with cold phosphate-buffered saline (PBS) containing 0.1% BSA and 0.02% sodium-azide (NaN_3_). All further steps were performed on ice and all centrifugation steps at 4°C. Anti-Nicastrin antibodies were added and incubated on an orbital shaker at 4°C for 1 hour. Cells were washed three times in cold PBS + BSA + NaN_3_. The secondary, species-specific anti-rabbit (rat) IgG-FITC-conjugated Abs was added at 1:200 dilution for 1 hour, and incubated on the orbital shaker in the dark. Cells were washed twice in PBS + BSA + NaN_3_ and finally resuspended in 400 μl of PBS. Immunofluorescent staining acquisition and analysis of 10,000 gated, live cells was done using FACSCanto II (Becton-Dickinson, Erembodegem, Belgium) and FlowJo software, Version 7.1 (Tree Star, Inc., Ashland, OR, USA).

### siRNA

Small interfering RNA (siRNA) against Nicastrin were previously described (Lombardo *et al*.). siRNA against Notch4 (Gene Solution siRNA gene ID4855: Hs-Notch4-1 S100065170, Hs-Notch4-3 S100065184, Hs-Notch4-5 S102633533, Hs-Notch4-6 S102633540) were purchased from Qiagen (Hilden, Germany) and pooled together. Silencer green fluorescent protein (GFP) from Ambion (Invitrogen, Carlsbad, CA, USA) (AM4626) was used as negative control. Both mRNA and protein levels were tested to ensure knockdown efficacy. TAM-R cells were seeded at 1.5 × 10^5^ cells per well in 6-well plates and transfected the following day with 50 nM siRNA using Hyperfect transfection reagent (Qiagen 301705). For Nicastrin knockdown, cells were analysed 72 hrs later. For Notch4 knockdown, cells were harvested after 72 hrs and 2 × 10^5^ cells were reseeded. Cells were transfected on the same day with 50 nM siRNA. The samples were analysed 72 hrs later (total knockdown 144 hrs).

### RNA extraction and real-time PCR

Total RNA was extracted using RNeasy columns (Qiagen), and cDNA was reverse transcribed from 2 μg RNA using Applied Biosystems technology (Invitrogen) (AB 4368814). Quantitative real-time RT-PCR (qRT-PCR) was performed in triplicate using SYBR Green mix (Invitrogen 4309155) on SDS7900HT FAST detection system (Applied Biosystems). For the primer sequence refer to Table S1 in Additional file 1. Samples underwent three-step amplification at 94°C (1 min), 60°C (30 sec), 72°C (30 sec). Melting curves were analysed after 40 cycles. The Ct values for test genes were normalized to ACTB and relative expression represented as 2^-ΔΔCt^. To measure microRNA (miRNA) 200c expression levels, total RNA extracted was used to perform RT-qPCR using Taqman mature miRNA primers and probes (Applied Biosystems). Briefly, mature miRNA expression was measured using stem-loop reverse transcriptase primers for miRNA cDNA synthesis followed by Taqman PCR analysis. RNA was reverse transcribed followed by qPCR on a 7900 HT Fast Real-Time PCR System (Applied Biosystems). Duplicate samples and an endogenous control (small nuclear RNA (snRNA) U6) were used throughout. Expression levels of each miRNA were evaluated using comparative threshold cycle (Ct) method using the 2^-ΔΔCt^ method with normalization to U6.

### Cell lysis and western blot

Briefly, cells were washed twice in ice-cold PBS and lysed in RIPA (Sigma-Aldrich R02780) buffer supplemented with Protease (Roche 11697498001) and phosphatase inhibitors (Sigma-Aldrich 93482) for 10 minutes in ice with intermittent vortexing. Samples were cold-centrifuged at maximum speed for 15 minutes and supernatant transferred to a clean cold eppendorf. The protein concentration of each sample was determined using a bicinchoninic acid (BCA) assay (Thermo Fisher Scientific, Waltham, MA, USA, 23227). Equal amounts of lysates were subject to immunoblotting on SDS-PAGE. Proteins were transferred to a Biotrace NT membrane (VWR, Radnor, PA, USA, PN66485) and incubated with primary antibodies. Proteins were visualized using donkey anti-mouse, and anti-rabbit secondary antibodies conjugated to the IRDyes, IR680-LT (926-68022) or IR800 (926-32213) (LI-COR Biosciences, Lincoln, NE, United States) and the LI-COR Odyssey 2.1 system. 16-bit images were analysed and quantified using the Odyssey analysis software.

### Sulforhodamine B colorimetric, and Transwell assay

Sulforhodamine B (SRB, Sigma-Aldrich S9012) colorimetric assay was used to assess cell proliferation and has been previously described (Lombardo *et al*.). For drug resistance experiments, cells were seeded at 3,000 per well in a 96-well plate and allowed to adhere overnight. Time 0 plate was fixed at this time, further plates were treated with 10^-7^ M 4-hydroxy-tamoxifen (4-OH-tamoxifen) and assayed at two-day intervals. For knockdown experiments, cells were seeded as in knockdown experiments, after 72 hours cells were harvested and subject to SRB protocol just described.

Transwell (VWR, 3422) cell invasion assay was used to assess cell invasive/migratory capacity. 2 × 10^5^ cells were pre-treated as previously described and seeded on a 6-well plate. Fifty-four hours later, cells were harvested and counted. A total of 50,000 cells were washed and resuspended in 200 μl αMEM and seeded in duplicate on the matrigel-coated (Becton Dickinson, Franklin Lakes, NJ, USA, 356237) Transwell upper chamber to assess invasion. To determine migration capacity, matrigel was excluded. FCS DMEM or DCFCS DMEM was used as chemoattractant for MCF7 and TAM-R respectively. Eighteen hours later, cells that did not migrate were wiped off from the top chamber with a cotton swab, the bottom chamber was fixed with 4% PFA (VWR P38), washed twice in PBS, cut and mounted on coverslips coated with Mowiol (Applichem, Darmstadt, Germany, A9011) infused with DAPI (Lonza, Basel, Switzerland, PA-3013). Ten pictures per condition were taken using an EVOS microscope system (Advanced Microscopy Group, Bothell, WA, USA). Fifty different fields were counted.

### Crystal violet staining

The cells were seeded on a 6-well plate and the pictures were taken at 50% confluence. Cells were washed twice in 1X PBS. 1 ml of Crystal Violet (100 mg Crystal Violet, Sigma-Aldrich, 20 mls ethanol, 80 mls dd-H2O) was added to the well for 30 minutes. Cells were washed 5X in PBS and pictures were taken using Axiovert system microscope (Carl Zeiss, Oberkochen, Germany).

### CD24/CD44 sorting

FACS sorting experiment was previously described (Lombardo *et al*.). Cells were pre-treated and seeded in 6-well plates for 72 hrs.

### Mammosphere formation assay and fractionation

Following MAbs/GSI pre-treatment, TAM-R cells were plated at single cell suspension at a density of 1,000 viable cell/well in ultralow attachment 24-well plates (Corning Life Sciences, Tewksbury, MA, USA). Cells were grown in a serum-free mammary epithelial growth medium (MEBM) (Lonza) supplemented with B27 (Invitrogen), 20 ng/ml EGF, 20 ng/ml bFGf (BD Biosciences, San Jose, CA, USA), and 4 μg/ml heparin (Sigma-Aldrich). Mammospheres were grown for 10 days and phase contrast images were taken using Axiovert system microscope (Carl Zeiss). For the second generation experiment, first generation mammospheres were collected and spun at 500 × g per 5 minutes. The pellet was resuspended in 50 μl Trypsin and the sample was passed 25 times through a sterile needle to get single cell suspension The same density of cells as in first generation culture was seeded, and cells were allowed to grow for 10 days.

Cytoplasm, membrane, and nucleus were fractioned using Subcellular Protein Fractionation kit from Thermo Fisher Scientific (78840) according to the manufacturer protocol. Adherent and mammospheres MCF7 and TAM-R were lysed in parallel. 10 μg of each fraction were loaded onto SDS-PAGE gel.

### Immunofluorescence

Cells were fixed in pre-warmed 4% paraformaldehyde/PBS and permeabilized in 0.2% Triton-X-100/PBS for 10 minutes. Cells were stained with anti-E-cadherin antibody (HECD-1) (M106, Takara Bio Inc., Shiga, Japan) and visualised with Alexa Fluor 488. Nuclei were counterstained with DAPI (Lonza, PA-3013). Images were taken using Axiovert system microscope (Carl Zeiss).

### Statistical analysis

Two-tailed Student *t* test was employed to determine the statistical significance of the differences observed. A *P* value below 0.05 was considered significant.

## Results

### ETR breast cancer cells display migratory behaviour, high levels of EMT-related genes, Nicastrin and Notch4

In our study, we used two alternative models of acquired ETR: 1) tamoxifen-resistant (TAM-R) cells obtained from MCF7 cells cultured with 4-OH-TAM over a period of 12 months as previously described [3]; 2) MCF7 LTED cells, which gradually acquired estrogen independence upon culture in estrogen/steroid-free conditions modelling aromatase inhibitor resistance [7].

**Figure 1:**
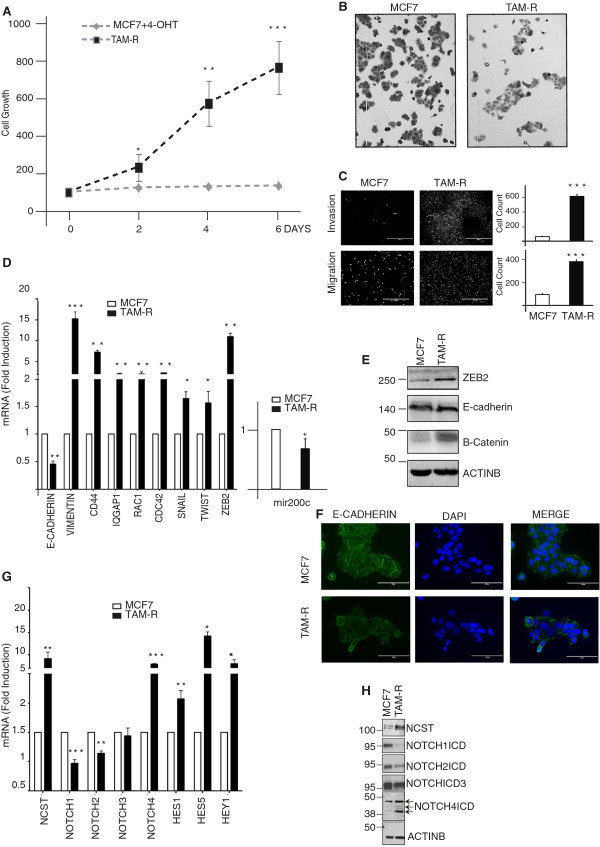
**Tamoxifen-resistant cells (TAM-R) are resistant to tamoxifen (4-OH-TAM), phenotypically distinct, more invasive and migratory compared to wild-type MCF7. (A)** MCF7 and TAM-R cells treated with vehicle (EtOH) or 10^-7^ M 4-OH-TAM were plated (3 × 10^3^/well) in 96-well plates and allowed to adhere. One plate was fixed and annotated as Day 0. A sulforhodamine B (SRB) assay was performed every two days until Day 6. The experiment was repeated three times and each time six technical replicates were used. **(B)** Cells were stained with Crystal Violet and 10X images were taken with a bright-field microscope when cells were 50% confluent (bar represents 400 μm). **(C)** Boyden chamber-based assay was used to determine the cells invasive or migratory capacity. Cells were allowed to invade or migrate for 72 or 18 hrs respectively before the insert was fixed, cut, and mounted in Mowiol infused with DAPI. 4X images were taken with a fluorescent microscope (bar represents 1,000 μm). The results are representative of three biological and two technical replicates. **(D)** Quantification of microRNA (mRNA) levels of epithelial to mesenchymal transition (EMT) markers or Notch genes **(G)** analysed by qRT-PCR. Fold change is shown in TAM-R compared to MCF7 cells, normalised to GAPDH. Results represent three biological as well as three technical replicates of each. (Bars represent standard deviation (SD) ^*^P <0.05 ^**^P <0.01, ^***^P <0.0001, *t* Student, two-tails). **(E)** Western blot validation for representative EMT markers and Notch proteins **(H)**. ActinB was used as loading control. **(F)** Representative images showing E-cadherin expression in MCF7 and TAM-R cells at cell-cell contact.

We first demonstrated that ETR cells were able to proliferate in estrogen-deprived medium and in presence of 4-OH-TAM as opposed to the parental MCF7 cells that showed growth arrest in the same conditions (Figure 1A, Figure S1A in Additional file 2 and Figure S2A in Additional file 3). Furthermore, we observed that although MCF7 cells grew in clusters and showed a typical epithelial cobblestone appearance, ETR cells displayed elongated, irregular morphology (Figure 1B, Figure S1B in Additional file 2). These changes in morphology suggested that ETR cells have undergone the EMT process that led to the acquisition of invasive and migratory capacity (Figure 1C, Figure S1C in Additional file 2). We further confirmed that these cells displayed reduced mRNA levels of E-cadherin, a cell-cell adhesion protein usually lost during EMT process, and increased levels of important EMT-related genes such as Vimentin, CD44, IQGAP1, RAC1, CDC42, SNAIL, TWIST, ZEB2 and mir200c (Figures 1D and Figure S1D in Additional file 2). We confirmed by western blot analysis that ZEB2 and B-catenin were upregulated. E-cadherin total expression levels were not significantly reduced. However, we observed a reduction of E-cadherin expression at cell-cell contact in the resistant cells (Figure 1F). To determine whether the Notch pathway is involved in ETR invasive features, we first assessed the levels of Notch receptors, Notch targets and Nicastrin, previously described as an important regulator of EMT and breast cancer stem cells [13].

Western blot and qRT-PCR showed that ETR cells displayed increased levels of Nicastrin and Notch targets (Hes1, Hey1 and Hes5). Interestingly, we also found lower mRNAs levels of Notch1, Notch2 and higher levels of Notch4 (Figure 1G). By western blot analysis we evaluated the expression of Notch intracellular domain (ICD) (Figure 1H). Notch activation was confirmed by EDTA treatments (Figure S2b in Additional file 3). We found that ETR cells displayed lower levers of Notch1 and Notch2 intracellular domains (N1ICD and N2ICD). By contrast, ETR cells displayed an increase activation of Notch4 (N4ICD), suggesting a strong signalling through this receptor (Figures 1G-H, Figure S1E-F in Additional file 2).

### Targeting NCT/Notch4 signalling induces reversal of EMT in ETR cells

**Figure 2:**
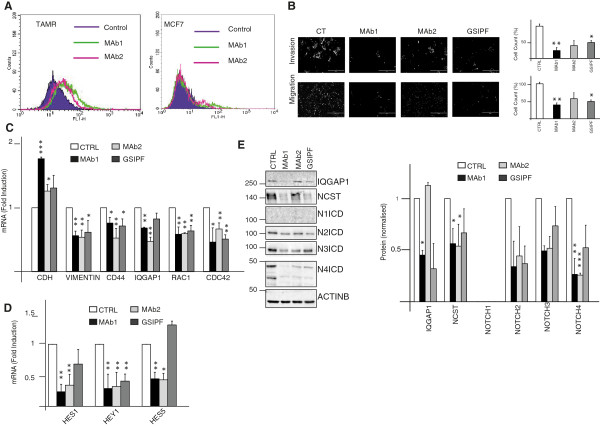
**Nicastrin (NCST) monoclonal antibodies (mAbs) and gamma secretase inhibitor (GSI) Pfizer can reverse epithelial to mesenchymal transition (EMT) process. (A)** Binding of anti-NCST mAbs to endogenous cells surface Nicastrin of tamoxifen-resistant (TAM-R) and MCF7 cells. Non-permeabilised cells were incubated with 50 μg/ml of mAbs1/2, followed by incubation with the secondary anti-rat FITC antibody. Rat immunoglobulin G (IgG) was used as control. Binding was assessed by FACS. **(B)** TAM-R cells were pre-incubated for 30 minutes with 50 μg/ml of mAb1/2, or 10 μM GSIPF (PF03084014). For invasion assay, cells were seeded in the upper compartment on top of matrigel-coated membrane and allowed to invade for 72 hrs. For motility assay, matrigel was excluded. Pre-treated cells were seeded on 6-well plates for 54 hrs, then harvested and counted. A total of 50,000 were transferred to the upper compartment for 18 hrs. The results are representative of three biological and two technical replicates. **(C, D)** qRT-PCR showing mAbs and GSIPF treatment modulate EMT and Notch-responsive genes. Cells were treated as in B and analysed 72 hrs later. microRNA (mRNA) levels are represented as fold induction normalised to GAPDH and compared to control. Results represent three biological as well as three technical replicates of each. **(E)** Representative western blot showing downregulation of Nicastrin and Notch proteins following mAbs and GSIPF treatments. IQGAP1 was chosen as EMT representative protein. Quantitation represents the average from three biological experiments (bars represent standard deviation (SD)).

**Figure 3:**
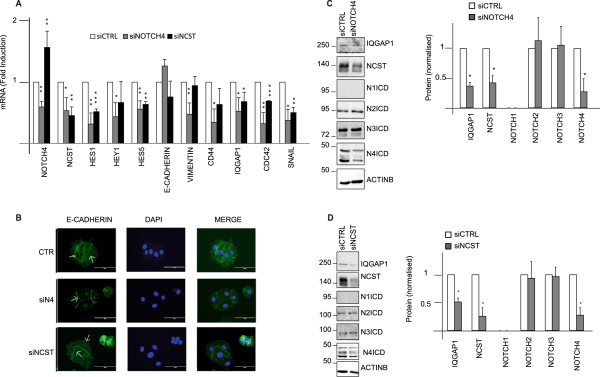
**Notch4 and Nicastrin knockdown reduce epithelial to mesenchymal transition (EMT) genes expression. (A)** qRT-PCR showing Notch4 and Nicastrin knockdown effect on EMT as well as Notch-responsive genes. microRNA (mRNA) levels are normalised to GAPDH. (n = 3 bars represent standard deviation (SD) ^*^P <0.05.) **(B)** Representative images showing E-cadherin expression in MCF7 after control, Notch4 or Nicastrin knockdown. **(C)** Representative western blot confirming specificity for small interfering RNA (siRNA) targeting Notch4. IQGAP1 was chosen as EMT representative protein (n = 3, bars represent SD ^*^P <0.05). **(D)** Representative western blot confirming Nicastrin siRNA effect on Notch receptors and IQGAP1 (n = 3, bars represent SD ^*^P <0.05).

To investigate the role of Nicastrin and Notch4 in ETR cells, which showed EMT features, we examined the effects on cell invasion or migration of two anti-Nicastrin mAbs (mAb1 and mAb2) that specifically bind Nicastrin at the cell surface of TAM-R cells compared to MCF7 cells (Figure 2A) and thereby block Notch signalling. We compared these results with two different GSIs (PF03084014, and RO4929097). Invasion and migration were significantly inhibited by mAb1, and so all the EMT genes. mAb2 was less effective in inhibiting migration and invasion behaviour but still effective in reducing EMT markers. Lastly, GSIPF was as potent as mAb1 in affecting migration and invasion and most of the EMT genes (Figure 2B-C, Figure S3A-B in Additional file 4). However, RO4929097 (GSIRO) did not show a significant effect (Figure S3A-D in Additional file 4 and Figure S3F in Additional file 4). E-cadherin mRNA expression levels significantly increased after mAb1 and mAb2. Importantly, all the treatments partially induced E-cadherin re-localisation to the cell-cell contact in TAM-R cells. (Figure S4A in Additional file 5) Upon mAb1, mAb2 treatments we observed a reduction of Hes1, Hey1, and Hes5, while GSIPF treatment was able to reduce Hey1mRNA expression levels (Figure 2D, Figure S3E in Additional file 4). Concomitantly, efficacy was further confirmed by reduction of Notch2, Notch3 and Notch4 activation (Figure 2E, Figure S3E in Additional file 4). Interestingly, we observed decreased levels of Nicastrin (Figures 2E). GSIRO efficiently attenuated the cleavage of Notch1, Notch2, Notch3, and Notch targets expression. However, it increased Notch4 and Nicastrin expression (Figures S3F in Additional file 4). To further validate the role of Nicastrin and Notch4 in mediating ETR cells behaviour, we depleted Nicastrin and Notch4 using specific siRNAs in TAM-R cells. siRNA Notch4 deconvolution is shown in Figure S2C in Additional file 3. We pooled the oligos together for further analyses. Nicastrin siRNAs were used as previously described [13,20]. siRNA against Notch4 were effective in reducing the expression of different Notch targets (Hes1, Hey1, Hes5) as well as EMT genes (Figure 3A and C). siRNA against Nicastrin efficiently reduced Hes1, Hes5 and most of the EMT genes (Figure 3A-D). Moreover, we confirmed a partial E-cadherin re-localisation to the cell-cell contacts in TAM-R cells upon siRNA (Figure 3B).

These data clearly suggest that the downregulation of Nicastrin and Notch4 expression in ETR cells could reverse the EMT phenotype.Surprisingly, following Notch4 depletion we observed a reduction of Nicastrin both at protein and mRNA levels (Figure 3A-C). As expected, Nicastrin knockdown decreased Notch4 cleavage as a consequence of GS activity inhibition (Figure 3D), suggesting a positive feedback mechanism between Notch4 and Nicastrin.

### Nicastrin and Notch4 regulates stem cell content and properties in ETR cells

**Figure 4:**
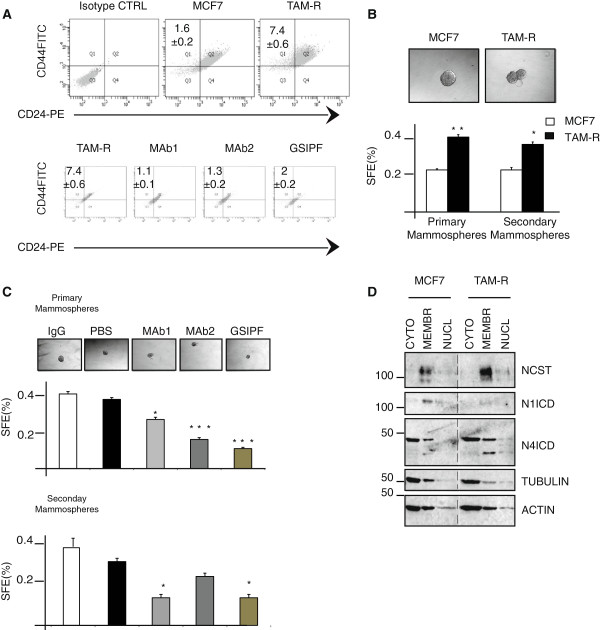
**Stem cell content population is increased in tamoxifen-resistant (TAM-R) cells and is affected by anti-Nicastrin (NCST) monoclonal antibodies (mAbs) and gamma secretase inhibitor (GSI) treatment. (A)** Percentage of CD44^+^/CD24^-^ cells assessed by FACS in TAM-R compared to MCF7 cells. The graph shows CD44^+^/CD24^-^ percentage assessed by FACS in TAM-R cells pre-treated with mAbs or GSIPF as in 2B. All treatments reduce the stem content population. **(B)** TAM-R cells form more primary and secondary mammospheres. Sphere formation efficacy (SFE) was calculated as the number of spheres formed in 10 days from the original number of single cells seeded and expressed as percentage. Bars represent mean percentage of mammospheres ± standard deviation (SD) from five separate replicates and three separate experiments. A representative image is shown at 10X magnification (^*^P <0.05, ^**^P <0.01). **(C)** SFE in secondary mammospheres is greatly reduced in mAb1 and GSIPF-treated cells. A representative image of mammospheres is shown as in **B** (bars represent SD) ^*^P <0.05, ^**^P <0.01) **(D)** MCF7 and TAM-R stem cells were fractionated into cytoplasm, membrane and nucleoplasm. Lysates were immunoblotted and probed for NCST, Notch1, and Notch4. Tubulin was used as cytoplasmic marker. ActinB was used to ensure each fraction was loaded equally.

We next examined whether the altered expression of EMT-related genes in TAM-R cells correlated with changes in CD44 and CD24 expression, reflecting an effect on the stem cell population. TAM-R cells showed a higher content of stem-like cells defined by the CD44^+^/CD24^-^ phenotype compared to MCF7 cells (Figure 4A). Consistent with this, the mammosphere formation assay showed that TAM-R formed more primary and secondary mammospheres than parental MCF7 cells (Figure 4B). We found that the percentage of CD44^+^/CD24^-^ cells significantly decreased after mAb1, mAb2 and GSIPF treatments (Figure 4A). Importantly, all the treatments reduced the sphere-forming efficiency (SFE) at the first generation, both mAb1 and GSIPF affected the self-renewal as demonstrated by the reduced SFE at the second generation of mammospheres (Figure 4C). Finally, MCF7 and TAM-R derived mammospheres were fractionated into cytoplasm, membrane and nucleoplasm. We found that, TAM-R-derived mammospheres expressed higher levels of Nicastrin in the membrane fraction, whereas Notch4 was increased in both membrane and nuclear fractions (Figure 4D), implying a role of these two proteins in the putative ETR stem cell population.

### Overexpression of Nicastrin confers tamoxifen resistance through Notch4 activation

**Figure 5:**
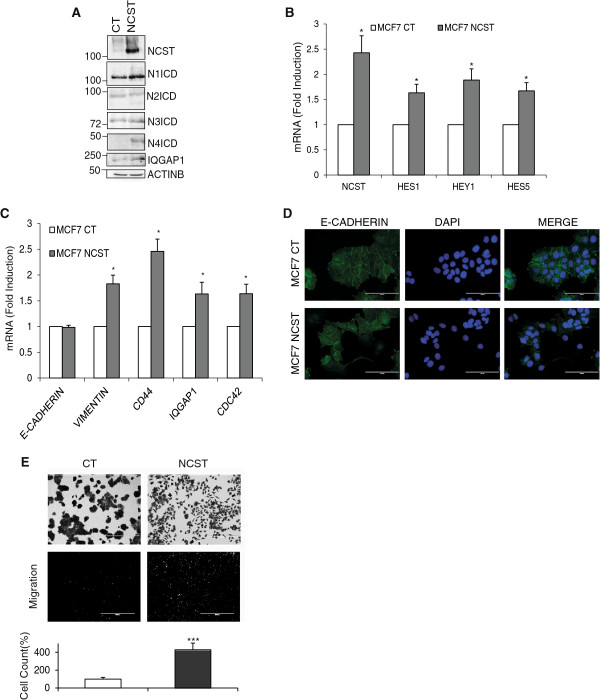
**Stable Nicastrin (NCST) overexpression activates Notch pathways and epithelial to mesenchymal transition (EMT) in MCF7 cells. (A)** Representative western blot of NCST, Notch receptors and IQGAP1 levels in control (CT) and NCST overexpressing MCF7 cells. ActinB was used as loading control. **(B)** qRT-PCR showing Nicastrin overexpression effect on Notch1, Notch4, Notch-responsive genes, and EMT genes **(C)**. microRNA (mRNA) levels were quantified relative to GAPDH. The mean of three different experiments is shown. **(D)** Representative images showing E-cadherin expression in MCF7 CT and MCF and NCST cells at cell-cell contact. **(E)** CT and MCF7 NCST cells were stained with Crystal Violet and 10X images were taken with bright-field microscope when cells were 50% confluent (bars represent 400 μM). Boyden chamber-based assay was used to determine the migratory capacity of MCF7 NCST compared to MCF7 CT cells. Cells were allowed to migrate for 18 hrs before the insert was fixed, cut, and mounted in Mowiol infused with DAPI. 4X images were taken with a fluorescent microscope. (Bars represent 1,000 μM). The results are representative of three biological and two technical replicates.

**Figure 6:**
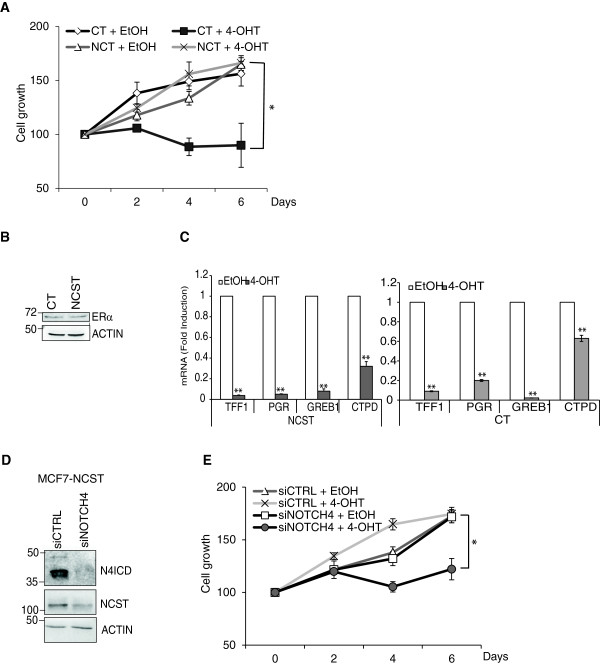
**Stable Nicastrin (NCST) overexpression confers 4-OH-TAM resistance in MCF7 cells through Notch4 activation. (A)** MCF7 CT and MCF7 NCST cells treated with vehicle (EtOH) or 10^-7^ M 4-OH-TAM were plated (3 × 10^3^/well) in 96-well plates and allowed to adhere. One plate was fixed and annotated as Day 0. A sulforhodamine B (SRB) assay was performed every two days until Day 6. The experiment was repeated three times and each time six technical replicates were used. **(B)** Representative western blot of estrogen receptor alpha (ERα) protein levels in MCF7 CT and MCF7 NCST cells. ActinB was used as loading control. **(C)** Quantification of microRNA (mRNA) levels of ERα target genes analysed by qRT-PCR. Fold change is shown in MCF7 NCST compared to MCF7 CT cells, normalised to GAPDH. Results represent three biological as well as three technical replicates of each. **(D)** Representative western blot confirming Notch4 small interfering RNA (siRNA) in MCF7 NCST cells and the effect on NCST protein. **(E)** An SRB assay performed as in A following siRNA transfection in MCF7 NCST. The experiment was repeated three times and each time six technical replicates were used.

To further investigate the contribution of Nicastrin in ETR cells, we stably overexpressed Nicastrin in MCF7 cells (MCF7 NCST). Nicastrin overexpression was confirmed at the protein and mRNA levels (Figure 5A-B). Nicastrin overexpression induced activation of Notch1, Notch3 and Notch4 (Figure 5A). Moreover, Notch targets levels (Hes1, Hey1 and Hey5) were increased (Figure 5B). We also observed a significant increase in EMT genes (Vimentin, CD44, IQGAP1, and CDC42) consistent with the morphological alteration and migration behaviour of Nicastrin overexpressing cells (Figure 5A,C-E). E-cadherin mRNA levels did not significantly change in NCST overexpressing cells. However, by immunofluorescence, we observed a reduction of E-cadherin expression at cell-cell contact in the these cells (Figure 5D).Finally, since ETR cells displayed high levels of Nicastrin, we evaluated whether Nicastrin can induce tamoxifen resistance. As shown in Figure 6A, Nicastrin overexpression was able to make MCF7 cells unresponsive to 4-OH-TAM. Importantly, ERα protein levels did not change in MCF7 NCST cells (Figure 6B). In addition, ERα target genes (TTF1, PGR, GREB1 and CTPSD) were efficiently reduced by 4-OH-TAM treatment in both control and NCST cells (Figure 6C). These data suggest that Nicastrin leads to an EMT phenotype through the activation of the Notch pathway and this can enable cancer cells to escape anti-estrogen treatments.

Importantly, when we depleted Notch4 by siRNA in MCF7 NCST cells, we observed downregulation of Nicastrin confirming the existence of a positive feedback between these two proteins (Figure 6D). Last, 4-OH-TAM response was restored in MCF7 NCST cells after Notch4 siRNA (Figure 6E). These results suggest a direct role of Notch4 in endocrine resistance due to Nicastrin. Moreover, expression data from publically available breast cancer patient dataset demonstrate that Notch4 expression significantly correlates with several markers of EMT transition. Indeed, Pearson correlation coefficient between RNA-seq data shows that high expression of Notch4 correlate with high expression of VIM, ZEB1/2 and SNAI1/2/3 while correlating with low expression of E-cadherin (CHD1) (Figure S5A in Additional file 6). Finally, we found a positive correlation between Notch4 expression and post-progression survival in ERα-positive breast cancer patients (Figure S5B in Additional file 6).

## Discussion

Endocrine therapies have been used for the treatment of ERα^+^ve breast cancers for over 30 years [23]; nonetheless the precise mechanisms behind the development of resistance are currently unknown.

In this study we demonstrate the key contributions of Notch4 and Nicastrin in the development of resistance to endocrine therapies. We used two different models of endocrine resistance: tamoxifen-resistant cells, obtained after culturing MCF7 cells with 4-hydroxy tamoxifen over a period of 12 months [3], and long-term estrogen-deprived cells, which have gradually acquired resistance after culture in estrogen/steroid-free conditions, modelling aromatase inhibitor resistance [7]. These *in vitro* systems reflect metastatic tumour behaviour and poor outcome often observed in patients that acquired endocrine resistance. For instance, both models display profound morphological and molecular changes, with cells showing EMT features and invasive/migratory capacity. EMT has been described as an important mechanism in tumour metastasis, allowing polarized epithelial cells to acquire a fibroblast-like phenotype permissive for intravasation and metastasis [24].

Activation of alternative survival signalling pathways can compensate for ER signalling inhibition following endocrine therapies [5,6]. Recently, the Notch pathway has been shown to be a significant factor in the development of endocrine resistance [7,8]. It has also been shown that combining GSI with tamoxifen causes tumour regression *in vivo*. Moreover, it has been demonstrated that Notch signalling is augmented in ETR cells following a global reprogramming of the epigenome [7]. Here, we show that ETR cells express high levels of Notch4 and Nicastrin, a key GS component responsible for the cleavage and the subsequent activation of Notch receptors. Importantly, ETR cells express lower levels of Notch1 and Notch2 compared to the endocrine-sensitive MCF7 cells suggesting a switch to Notch4 activation after acquiring endocrine resistance.

Notch receptors have been shown to be important for the formation of spontaneous mammary tumors *in vivo* after overexpression of constitutive, active forms of Notch1 or Notch4 [25]. Moreover, overexpression of constitutively active Notch4 in normal human mammary epithelial cells induces transformation *in vivo*[26].

Notch signalling has also been implicated in EMT where it modulates the cell-cell adhesion protein E-cadherin [27]. Accordingly, we found low mRNA levels of E-cadherin in ETR cells. Since protein levels did not significantly change, we explored E-cadherin cellular localization. We showed a distrupted E-cadherin at the cell-cell contacts, which was restored by GSI, anti-Nicastrin mAbs or Notch4 and Nicastrin siRNA. In addition, several EMT genes were affected after the treatments suggesting a partial EMT reversal.

We have previously shown that Nicastrin regulates the EMT process and stem cell content in triple-negative breast cancers partially through Notch1/Notch4 activation [13]. Moreover, a critical role of Notch-4 rather than Notch1 has been recently shown for the survival of tumour-initiating cells [28].

Increasing evidence suggests that breast CSCs contribute to endocrine resistance. CSCs may be intrinsically endocrine resistant because they do not express ERα while exhibiting high levels of mesenchymal genes [3]. Interestingly, ETR breast cancer cells express high levels of EGFR and other growth factor receptors that have been linked to normal breast stem cells [29]. In addition, it has been demonstrated that Notch receptors are present in mammary stem cells and progenitor cells, and are downregulated in differentiated cells [17].

Notch4 appears to be absolutely required for growth in three-dimensional cultures as the development of branching structures is completely blocked by anti-Notch4 antibodies or GSI [17] ETR cells expressing increased expression of growth factor receptors and increased Notch4 levels may reflect an increased proportion of CSCs selected by endocrine therapies. However, up to the present time the precise mechanism was unknown.

Here, we demonstrated that TAM-R cells contain a higher content of CD44^+^/CD24^-^ CSCs as they form a higher number of mammospheres *in vitro* compared to the parental MCF7 cells. Importantly, pharmacological inhibition of Notch4 and Nicastrin using GSI PF03084014 and anti-Nicastrin mAbs is sufficient to decrease the percentage of CD44^+^/CD24^-^ cells and the sphere-forming efficiency. These results suggest that endocrine-resistant tumours are driven by a stem cell-like population possessing higher levels of Nicastrin, activated Notch4 and EMT features.

Finally, we demonstrate that Nicastrin overexpression is sufficient to bypass tamoxifen response and is linked to acquisition of EMT phenotype. Nicastrin overexpression induces the EMT process in non-invasive MCF10A breast cells, partially through Notch1/Notch4 activation [13]. Moreover, Nicastrin may contribute to drug resistance in human embryonic kidney (HEK293) cells [30]. Therefore it seems likely that Nicastrin overexpression can overcome endocrine therapy through increased activation of the Notch pathway. Although Nicastrin overexpression induced activation of Notch1ICD and Notch3ICD beside Notch4ICD as a consequence of an increase activity of gamma secretase [13], we found that Notch4 inhibition was sufficient to restore tamoxifen sensitivity. This indicates a major role of Notch4 in Nicastrin-mediated resistance to tamoxifen.

## Conclusions

In summary, we demonstrated that Nicastrin and Notch4 are key molecules involved in resistance to endocrine therapy. Our data suggest that targeting Notch4 and Nicastrin is a potential approach to reverse endocrine resistance in breast cancer patients.

## Abbreviations

CSC: cancer stem cells; DCIS: ductal carcinoma *in situ*; DMEM: Dulbecco’s modified Eagle’s medium; EGFR: epidermal growth factor receptor; EMT: epithelial to mesenchymal transition; ER: estrogen receptor alpha; ETR: endocrine therapy resistance; FCS: fetal calf serum; GS: gamma secretase; GSI: gamma secretase inhibitor; HER2: human epidermal growth factor 2; IgG: immunoglobulin G; LTED: long-term estrogen-deprived; mAb: monoclonal antibody; miRNA: microRNA; NCST: Nicastrin; PBS: phosphate-buffered saline siRNA, small interfering RNA; TAM-R: tamoxifen-resistant.

## Competing interests

The authors declare that they have no competing interests.

## Authors’ contributions

YL and MF were involved in study design, carried out the majority of the practical experimentation and drafted the manuscript. AF and LM provided assistance with experimental design and manuscript preparation. VV assisted in invasion assays experimentation, western blotting and PCR experiments. RCC conceived of the study, and participated in its design and coordination and helped to draft the manuscript. All authors read and approved the final manuscript.

## Supplementary Material

Additional file 1: Table S1 Primer list. Click here for file

Additional file 2: Figure S1 Long-term estrogen-deprived (LTED) cells are resistant to tamoxifen (4-OH-TAM), phenotypically distinct, more invasive and migratory compared to wild-type MCF7. **(A)** MCF7 and LTED cells treated with vehicle (EtOH) or 10^-7^ M tamoxifen (4-OH-TAM) were plated (3 x 10^3^/well) in 96-well plates and allowed to adhere. One plate was fixed and annotated as Day 0. A sulforhodamine B (SRB) assay was performed every two days until Day 6. The experiment was repeated three times and each time six technical replicates were used. **(B)** Cells were stained with Crystal Violet and 10X images were taken with bright-field microscope when cells were 50% confluent (bars represent 400 μm). **(C)** Boyden chamber-based assay was used to determine the LTED cells migratory capacity. Cells were allowed migrate for 18 hrs before the insert was fixed, cut, and mounted in Mowiol infused with DAPI. 4X images were taken (bars represent 1,000 μm). The results are representative of two biological and two technical replicates. **(D)** Quantification of microRNA (mRNA) levels of epithelial to mesenchymal transition (EMT) markers or Notch genes **(E)** analysed by qRT-PCR. Fold change is shown in LTED compared to MCF7 cells, everything normalised to GAPDH. **(F)** Western blot validation for Nicastrin and Notch receptors. ActinB was used as loading control. Click here for file

Additional file 3: Figure S2 **(A)** MCF7 cells were treated with vehicle (EtOH) or 10^-7^ M tamoxifen (4-OH-TAM) were plated (3 x 10^3^/well) in 96-well plates and allowed to adhere. One plate was fixed and annotated as Day 0. A sulforhodamine B (SRB) assay was performed every two days until Day 6. The experiment was repeated three times and each time six technical replicates were used. **(B)** Western blot analysis of N1ICD, N2ICD, N3ICD and N4ICD after EDTA treatment in tamoxifen-resistant (TAM-R) cells. ActinB was used as loading control. **(C)** Multiple small interfering RNA (siRNA) for Notch4 was tested. Following knockdown, proteins were prepared from whole cell lysate and immunoblotted against Notch4. Quantitation normalised to ActinB is shown. Click here for file

Additional file 4: Figure S3 Anti-Nicastrin (NCST) monoclonal antibodies (mAbs) and gamma secretase inhibitors (GSIs) effect on long-term estrogen-deprived (LTED) and tamoxifen-resistant (TAM-R) cells. **(A)** Boyden chambers were used to determine cells migratory capacity. LTED cells were pre-incubated for 30 minutes with 50 μg/ml of mAb1/2, or 10 μM GSIPF (PF03084014) or GSIRO (RO4929097). Pre-treated cells were seeded on 6-well plates for 54 hrs, then harvested and counted. A total of 50,000 were transferred to the chamber upper compartment for 18 hrs before the insert was cut, fixed, rinsed and mounted on Mowiol-DAPI coverslips. 4X images were taken (bars represent 1,000 μm). The results are representative of two biological and two technical replicates. **(B)** RO4929097 has no effect on TAM-R migration activity. Cells were treated as in 2B. 10X images were taken (bars represent 400 μm) The results are representative of two biological and two technical replicates. **(C, D)** Cells were treated as in 2B, microRNA (mRNA) was prepared and transcript levels were determined relative to GAPDH by qRT-PCR (N = 3 independent experiments, bars show standard deviation (SD)). EMT and Notch-related genes are shown. **(E)** Representative western blot showing GSI RO treatment followed by NCST increase. Notch4 cleavage is increased (50 KDa) or unaffected. Total protein was normalised to Actin (N = 3 independent experiment, bars show SD). Click here for file

Additional file 5: Figure S4 Representative images showing E-cadherin localization in tamoxifen-resistant (TAM-R) cells treated with control immunoglobulin G (IgG), monoclonal antibody 1 (mAb1), monoclonal antibody 2 (mAb2) and gamma secretase inhibitor Pfizer (GSIPF). Click here for file

Additional file 6: Figure S5 **(A)** Pearson correlation coefficient between RNA-seq data shows that high expression of Notch4 correlate with high expression of VIM, ZEB1/2 and SNAI1/2/3 while correlating with low expression of E-cadherin (CHD1). **(B)** Kaplan-Meier model comparing post-progression survival in estrogen receptor alpha (ERα)-positive breast cancer patients showing Notch4 expression. Click here for file

## References

[B1] Davies C Godwin J Gray R Clarke M Cutter D Darby S McGale P Pan HC Taylor C Wang YC Dowsett M Ingle J Peto R Relevance of breast cancer hormone receptors and other factors to the efficacy of adjuvant tamoxifen: patient-level meta-analysis of randomised trials Lancet 2011 378 771 784 21802721 10.1016/S0140-6736(11)60993-8 PMC3163848

[B2] Osborne CK Schiff R Mechanisms of endocrine resistance in breast cancer Annu Rev Med 2011 62 233 247 20887199 10.1146/annurev-med-070909-182917 PMC3656649

[B3] Hiscox S Jiang WG Obermeier K Taylor K Morgan L Burmi R Barrow D Nicholson RI Tamoxifen resistance in MCF7 cells promotes EMT-like behaviour and involves modulation of beta-catenin phosphorylation Int J Cancer 2006 118 290 301 16080193 10.1002/ijc.21355

[B4] Zhou C Zhong Q Rhodes LV Townley I Bratton MR Zhang Q Martin EC Elliott S Collins-Burow BM Burow ME Wang G Proteomic analysis of acquired tamoxifen resistance in MCF-7 cells reveals expression signatures associated with enhanced migration Breast Cancer Res 2012 14 R45 22417809 10.1186/bcr3144 PMC3446379

[B5] Hutcheson IR Knowlden JM Madden TA Barrow D Gee JM Wakeling AE Nicholson RI Oestrogen receptor-mediated modulation of the EGFR/MAPK pathway in tamoxifen-resistant MCF-7 cells Breast Cancer Res Treat 2003 81 81 93 14531500 10.1023/A:1025484908380

[B6] Kurokawa H Lenferink AE Simpson JF Pisacane PI Sliwkowski MX Forbes JT Arteaga CL Inhibition of HER2/neu (erbB-2) and mitogen-activated protein kinases enhances tamoxifen action against HER2-overexpressing, tamoxifen-resistant breast cancer cells Cancer Res 2000 60 5887 5894 11059787

[B7] Magnani L Stoeck A Zhang X Lanczky A Mirabella AC Wang TL Gyorffy B Lupien M Genome-wide reprogramming of the chromatin landscape underlies endocrine therapy resistance in breast cancer Proc Natl Acad Sci U S A 2013 110 E1490 E1499 23576735 10.1073/pnas.1219992110 PMC3631697

[B8] O’Brien CS Farnie G Howell SJ Clarke RB Breast cancer stem cells and their role in resistance to endocrine therapy Hormones Cancer 2011 2 91 103 21761332 10.1007/s12672-011-0066-6 PMC10358078

[B9] Yun J Pannuti A Espinoza I Zhu H Hicks C Zhu X Caskey M Rizzo P D’Souza G Backus K Denning MF Coon J Sun M Bresnick EH Osipo C Wu J Strack PR Tonetti DA Miele L Crosstalk between PKCalpha and Notch-4 in endocrine-resistant breast cancer cells Oncogenesis 2013 2 e60 23917222 10.1038/oncsis.2013.26 PMC3759125

[B10] Rizzo P Miao H D’Souza G Osipo C Song LL Yun J Zhao H Mascarenhas J Wyatt D Antico G Hao L Yao K Rajan P Hicks C Siziopikou K Selvaggi S Bashir A Bhandari D Marchese A Lendahl U Qin JZ Tonetti DA Albain K Nickoloff BJ Miele L Cross-talk between notch and the estrogen receptor in breast cancer suggests novel therapeutic approaches Cancer Res 2008 68 5226 5235 18593923 10.1158/0008-5472.CAN-07-5744 PMC4445363

[B11] Hao L Rizzo P Osipo C Pannuti A Wyatt D Cheung LW Sonenshein G Osborne BA Miele L Notch-1 activates estrogen receptor-alpha-dependent transcription via IKKalpha in breast cancer cells Oncogene 2010 29 201 213 19838210 10.1038/onc.2009.323 PMC4976641

[B12] Hu YY Zheng MH Zhang R Liang YM Han H Notch signaling pathway and cancer metastasis Adv Exp Med Biol 2012 727 186 198 22399348 10.1007/978-1-4614-0899-4_14

[B13] Lombardo Y Filipovic A Molyneux G Periyasamy M Giamas G Hu Y Trivedi PS Wang J Yague E Michel L Coombes RC Nicastrin regulates breast cancer stem cell properties and tumor growth in vitro and in vivo Proc Natl Acad Sci U S A 2012 109 16558 16563 23012411 10.1073/pnas.1206268109 PMC3478621

[B14] Takebe N Warren RQ Ivy SP Breast cancer growth and metastasis: interplay between cancer stem cells, embryonic signaling pathways and epithelial-to-mesenchymal transition Breast Cancer Res 2011 13 211 21672282 10.1186/bcr2876 PMC3218933

[B15] Raouf A Zhao Y To K Stingl J Delaney A Barbara M Iscove N Jones S McKinney S Emerman J Aparicio S Marra M Eaves C Transcriptome analysis of the normal human mammary cell commitment and differentiation process Cell Stem Cell 2008 3 109 118 18593563 10.1016/j.stem.2008.05.018

[B16] Yalcin-Ozuysal O Fiche M Guitierrez M Wagner KU Raffoul W Brisken C Antagonistic roles of Notch and p63 in controlling mammary epithelial cell fates Cell Death Differ 2010 17 1600 1612 20379195 10.1038/cdd.2010.37

[B17] Dontu G Jackson KW McNicholas E Kawamura MJ Abdallah WM Wicha MS Role of Notch signaling in cell-fate determination of human mammary stem/progenitor cells Breast Cancer Res 2004 6 R605 R615 15535842 10.1186/bcr920 PMC1064073

[B18] Farnie G Clarke RB Spence K Pinnock N Brennan K Anderson NG Bundred NJ Novel cell culture technique for primary ductal carcinoma in situ: role of Notch and epidermal growth factor receptor signaling pathways J Natl Cancer Inst 2007 99 616 627 17440163 10.1093/jnci/djk133

[B19] Stylianou S Clarke RB Brennan K Aberrant activation of notch signaling in human breast cancer Cancer Res 2006 66 1517 1525 16452208 10.1158/0008-5472.CAN-05-3054

[B20] Filipovic A Gronau JH Green AR Wang J Vallath S Shao D Rasul S Ellis IO Yague E Sturge J Coombes RC Biological and clinical implications of nicastrin expression in invasive breast cancer Breast Cancer Res Treat 2011 125 43 53 20224929 10.1007/s10549-010-0823-1

[B21] Dong Y Li A Wang J Weber JD Michel LS Synthetic lethality through combined Notch-epidermal growth factor receptor pathway inhibition in basal-like breast cancer Cancer Res 2010 70 5465 5474 20570903 10.1158/0008-5472.CAN-10-0173

[B22] Hayashi I Takatori S Urano Y Miyake Y Takagi J Sakata-Yanagimoto M Iwanari H Osawa S Morohashi Y Li T Wong PC Chiba S Kodama T Hamakubo T Tomita T Iwatsubo T Neutralization of the gamma-secretase activity by monoclonal antibody against extracellular domain of nicastrin Oncogene 2012 31 787 798 21725355 10.1038/onc.2011.265 PMC4058788

[B23] Jordan VC Tamoxifen: a most unlikely pioneering medicine Nat Rev Drug Discov 2003 2 205 213 12612646 10.1038/nrd1031

[B24] Hanahan D Weinberg RA Hallmarks of cancer: the next generation Cell 2011 144 646 674 21376230 10.1016/j.cell.2011.02.013

[B25] Callahan R Raafat A Notch signaling in mammary gland tumorigenesis J Mammary Gland Biol Neoplasia 2001 6 23 36 11467450 10.1023/a:1009512414430

[B26] Gallahan D Jhappan C Robinson G Hennighausen L Sharp R Kordon E Callahan R Merlino G Smith GH Expression of a truncated Int3 gene in developing secretory mammary epithelium specifically retards lobular differentiation resulting in tumorigenesis Cancer Res 1996 56 1775 1785 8620493

[B27] Timmerman LA Grego-Bessa J Raya A Bertran E Perez-Pomares JM Diez J Aranda S Palomo S McCormick F Izpisua-Belmonte JC de la Pompa JL Notch promotes epithelial-mesenchymal transition during cardiac development and oncogenic transformation Genes Dev 2004 18 99 115 14701881 10.1101/gad.276304 PMC314285

[B28] Farnie G Clarke RB Mammary stem cells and breast cancer–role of Notch signalling Stem Cell Rev 2007 3 169 175 17873349 10.1007/s12015-007-0023-5

[B29] Pancholi S Lykkesfeldt AE Hilmi C Banerjee S Leary A Drury S Johnston S Dowsett M Martin LA ERBB2 influences the subcellular localization of the estrogen receptor in tamoxifen-resistant MCF-7 cells leading to the activation of AKT and RPS6KA2 Endocr Relat Cancer 2008 15 985 1002 18824559 10.1677/ERC-07-0240

[B30] Pardossi-Piquard R Dunys J Giaime E Guillot-Sestier MV St George-Hyslop P Checler F Alves da Costa C p53-dependent control of cell death by nicastrin: lack of requirement for presenilin-dependent gamma-secretase complex J Neurochem 2009 109 225 237 19187441 10.1111/j.1471-4159.2009.05952.x

