# Supplementary material for: Nicastrin and Notch4 drive endocrine therapy resistance and epithelial to mesenchymal transition in MCF7 breast cancer cells
Source: Breast Cancer Res. 2014 Jun 11;16(3):R62. doi: 10.1186/bcr3675 (PMC4095694; doi:10.1186/bcr3675)
Supplement: Additional file 1: Table S1 — Primer list. [file bcr3675-S1.docx]

| **PRIMER** | FORWARD | REVERSE |
| --- | --- | --- |
| **GAPDH** | 5’-tgaaggtcggagtcaacggatttt-3’ | 5’-gccatcgaatttgccatgggtgg-3’ |
| **NICASTRIN** | 5'-gccagcaaagagcttgagtt-3' | 5'-ctggggtcctcctcagtatg-3' |
| **NOTCH1** | 5′-gtgactgctccctcaacttcaat-3′ | 5′-agtgacggccactgtgacag-3′ |
| **NOTCH2** | 5’-ccagctgctactcacaggtga-3’ | 5’-ggtttgtgcctgagaaccatac-3’ |
| **NOTCH3** | 5-cctgtcttcctgggtttgag-3’ | 5’-cagaactggcctgtccactc-3’ |
| **NOTCH4** | 5'-ccaaccctgcgataatgcgag-3' | 5'-agtcatccgttgagaccctgc-3' |
| **HES1** | 5’-cacagaaagtcatcaaagcc-3’ | 5’-cagaatgtccgccttctc-3’ |
| **HEY1** | 5'-cgaggtggagaaggagagtg-3' | 5'-ctgggtaccagccttctcag-3' |
| **HES5** | 5'-gcccggggttctatgatatt-3' | 5'-gagttcggccttcacaaaag-3' |
| **E-CADHERIN** | 5’-gtcaaagtcctggtcctc-3’ | 5’-gattctgctgctcttgct-3’ |
| **VIMENTIN** | 5’-acattgagattgccacct-3’ | 5’-tccagattagtttccctcaag-3’ |
| **CD44** | 5’-agcaaccaagcggcaagaaa-3’ | 5’-gtgtggttgaaatggtgctg-3’ |
| **IQGAP1** | 5'-tggaaaggttgacttcacagaa-3' | 5'-gtaatgcggcttcatccact-3' |
| **RAC1** | 5’-agcttttgcggagattttga-3’ | 5’-cccgtgacactttcattcct-3’ |
| **CDC42** | 5’-cccatcggaatatgtaccaac-3’ | 5’-aggcttctgtttgttcttggc-3’ |

**Supplementary table 1**
