# Supplementary figures and images for: Nicastrin and Notch4 drive endocrine therapy resistance and epithelial to mesenchymal transition in MCF7 breast cancer cells
Source: Breast Cancer Res. 2014 Jun 11;16(3):R62. doi: 10.1186/bcr3675 (PMC4095694; doi:10.1186/bcr3675)

**S1A**

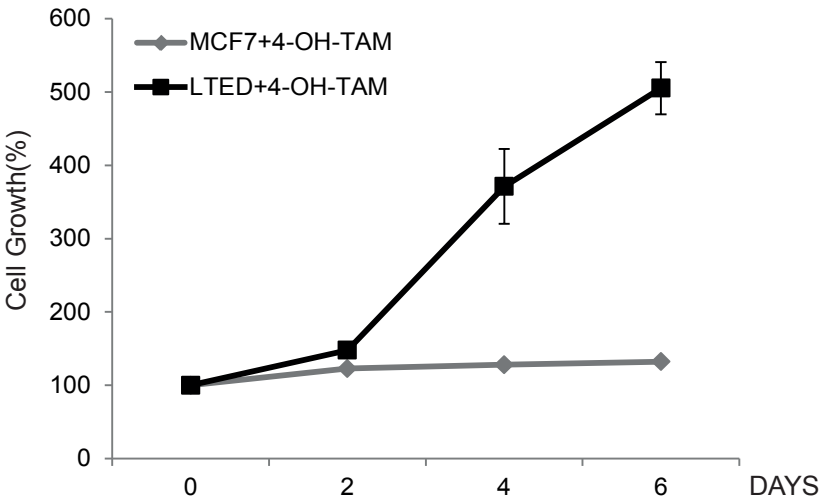

**S1B**

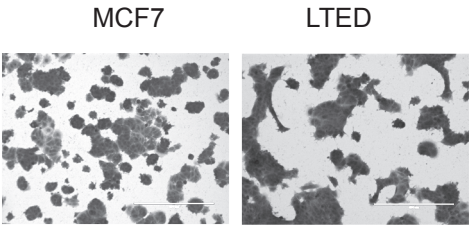

**S1C**

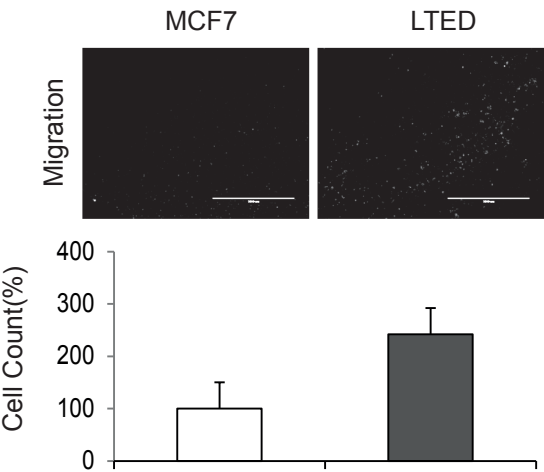

**S1D**

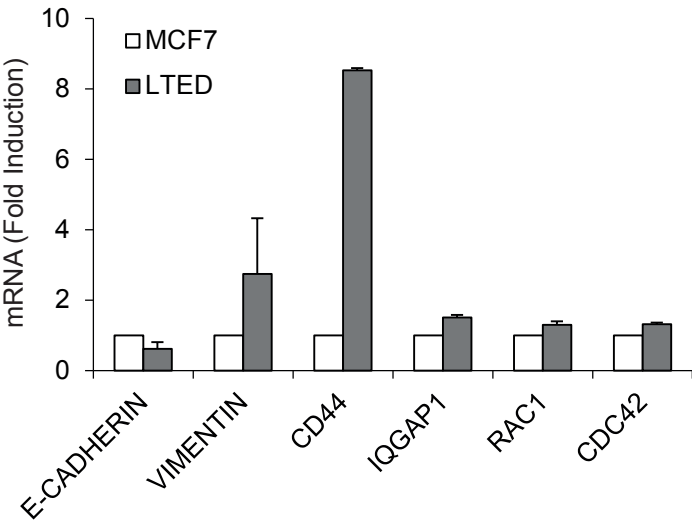

**S1E**

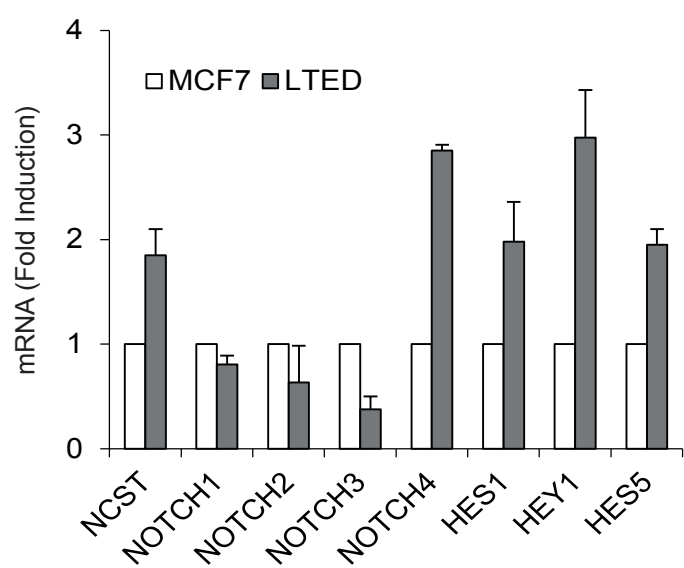

**S1F**

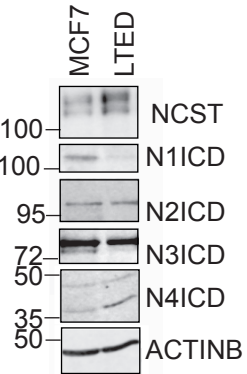

Supplement: Additional file 2: Figure S1 — Long-term estrogen-deprived (LTED) cells are resistant to tamoxifen (4-OH-TAM), phenotypically distinct, more invasive and migratory compared to wild-type MCF7. (A) MCF7 and LTED cells treated with vehicle (EtOH) or 10-7 M tamoxifen (4-OH-TAM) were plated (3 x 10^3^/well) in 96-well plates and allowed to adhere. One plate was fixed and annotated as Day 0. A sulforhodamine B (SRB) assay was performed every two days until Day 6. The experiment was repeated three times and each time six technical replicates were used. (B) Cells were stained with Crystal Violet and 10X images were taken with bright-field microscope when cells were 50% confluent (bars represent 400 μm). (C) Boyden chamber-based assay was used to determine the LTED cells migratory capacity. Cells were allowed migrate for 18 hrs before the insert was fixed, cut, and mounted in Mowiol infused with DAPI. 4X images were taken (bars represent 1,000 μm). The results are representative of two biological and two technical replicates. (D) Quantification of microRNA (mRNA) levels of epithelial to mesenchymal transition (EMT) markers or Notch genes (E) analysed by qRT-PCR. Fold change is shown in LTED compared to MCF7 cells, everything normalised to GAPDH. (F) Western blot validation for Nicastrin and Notch receptors. ActinB was used as loading control. [file bcr3675-S2.pdf]

S2A

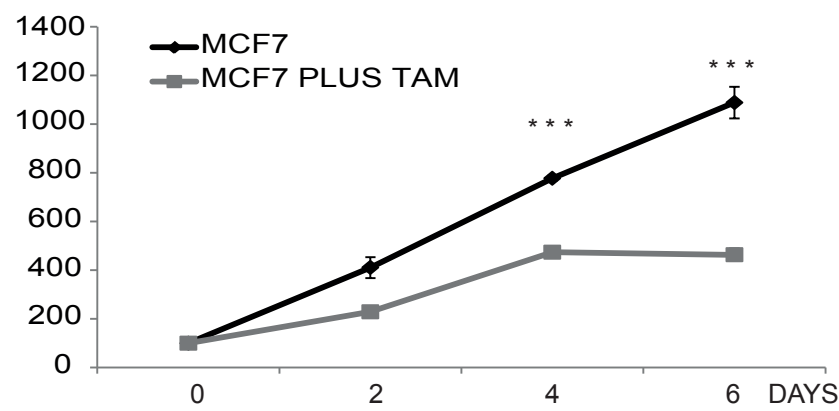

S2B

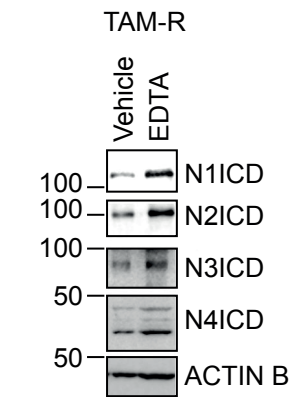

S2C

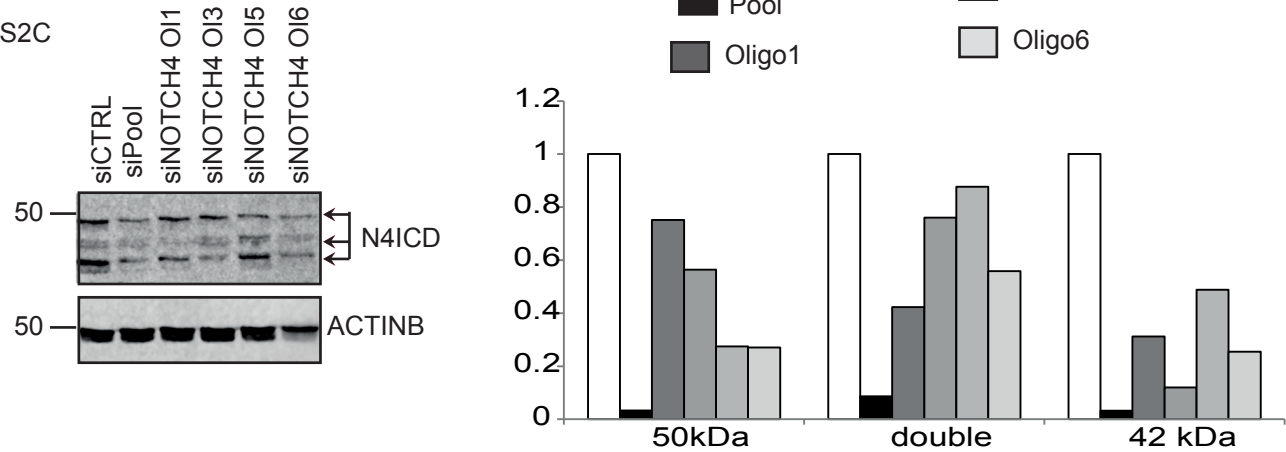

Supplement: Additional file 3: Figure S2 — (A) MCF7 cells were treated with vehicle (EtOH) or 10-7 M tamoxifen (4-OH-TAM) were plated (3 x 10^3^/well) in 96-well plates and allowed to adhere. One plate was fixed and annotated as Day 0. A sulforhodamine B (SRB) assay was performed every two days until Day 6. The experiment was repeated three times and each time six technical replicates were used. (B) Western blot analysis of N1ICD, N2ICD, N3ICD and N4ICD after EDTA treatment in tamoxifen-resistant (TAM-R) cells. ActinB was used as loading control. (C) Multiple small interfering RNA (siRNA) for Notch4 was tested. Following knockdown, proteins were prepared from whole cell lysate and immunoblotted against Notch4. Quantitation normalised to ActinB is shown. [file bcr3675-S3.pdf]

**S3A**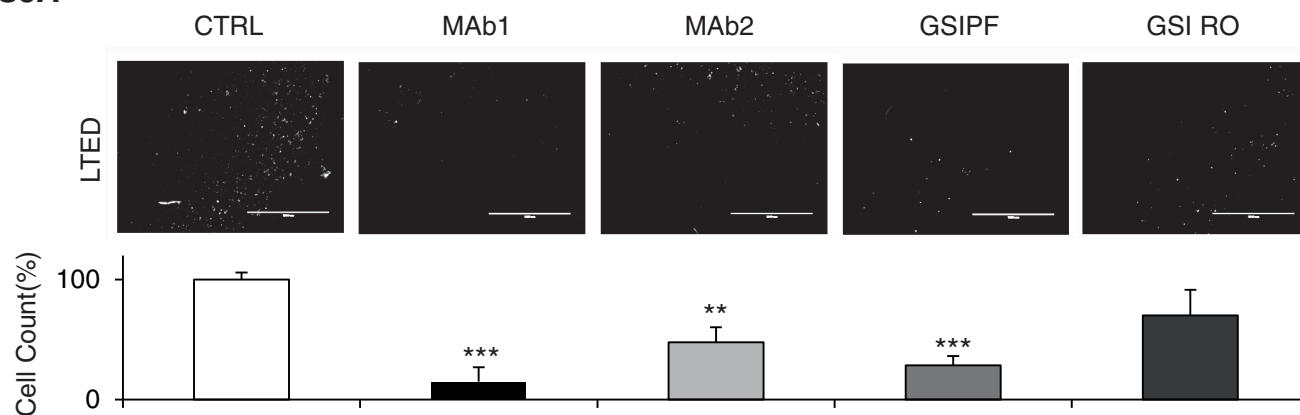**S3B**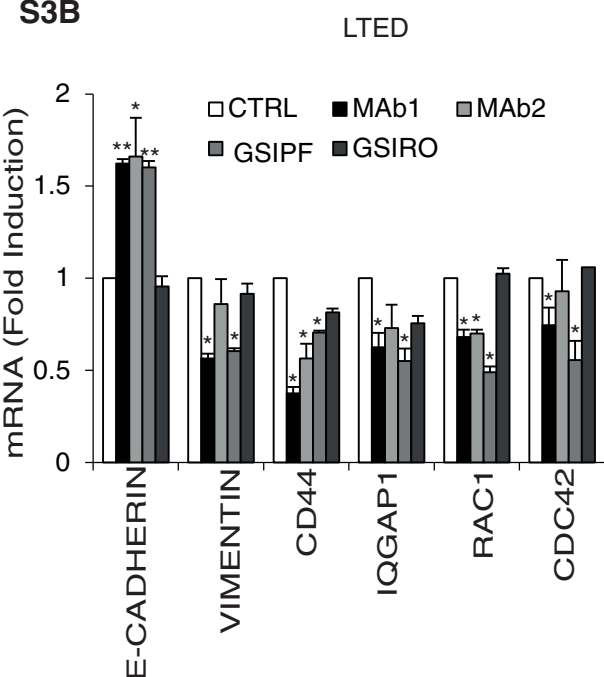**S3C**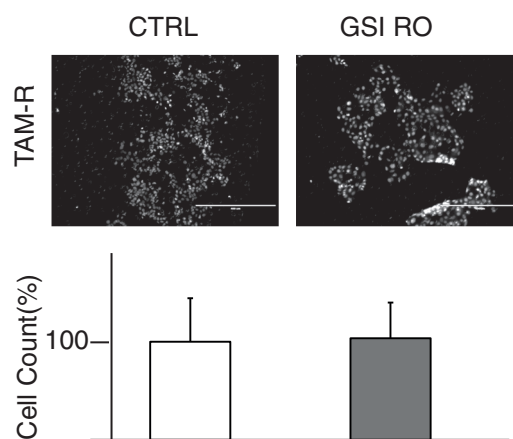**S3D**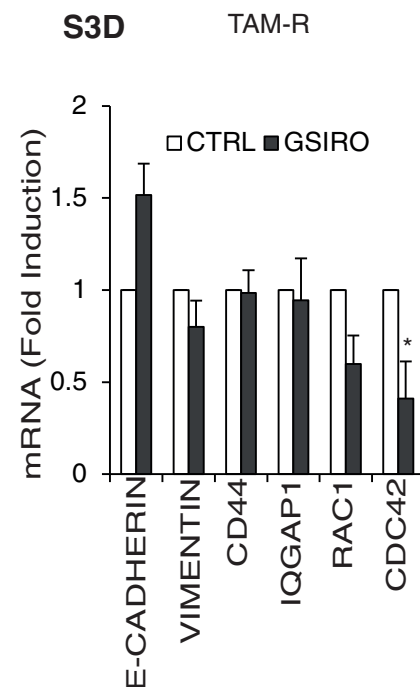**S3E**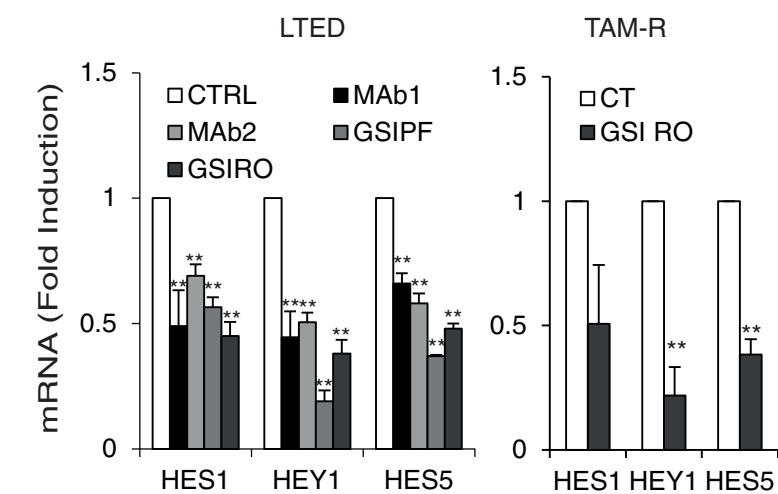**S3F**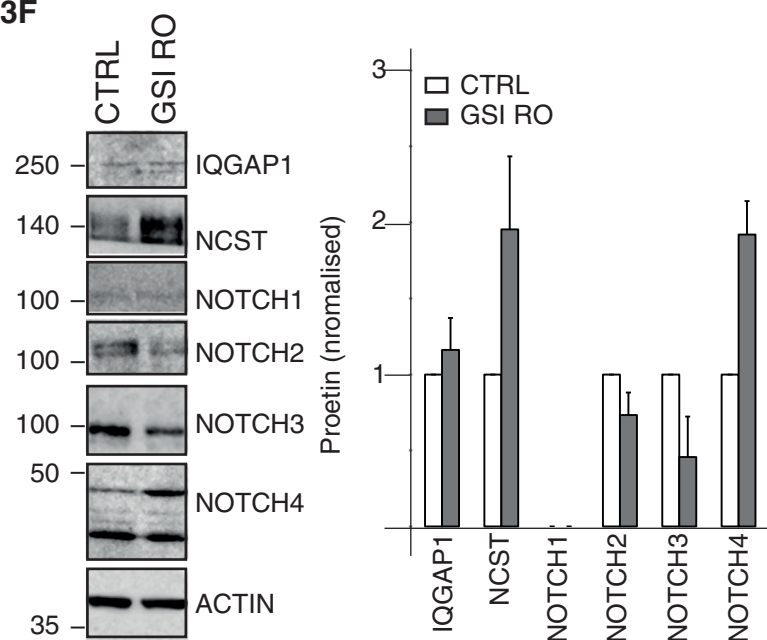

Supplement: Additional file 4: Figure S3 — Anti-Nicastrin (NCST) monoclonal antibodies (mAbs) and gamma secretase inhibitors (GSIs) effect on long-term estrogen-deprived (LTED) and tamoxifen-resistant (TAM-R) cells. (A) Boyden chambers were used to determine cells migratory capacity. LTED cells were pre-incubated for 30 minutes with 50 μg/ml of mAb1/2, or 10 μM GSIPF (PF03084014) or GSIRO (RO4929097). Pre-treated cells were seeded on 6-well plates for 54 hrs, then harvested and counted. A total of 50,000 were transferred to the chamber upper compartment for 18 hrs before the insert was cut, fixed, rinsed and mounted on Mowiol-DAPI coverslips. 4X images were taken (bars represent 1,000 μm). The results are representative of two biological and two technical replicates. (B) RO4929097 has no effect on TAM-R migration activity. Cells were treated as in 2B. 10X images were taken (bars represent 400 μm) The results are representative of two biological and two technical replicates. (C, D) Cells were treated as in 2B, microRNA (mRNA) was prepared and transcript levels were determined relative to GAPDH by qRT-PCR (N = 3 independent experiments, bars show standard deviation (SD)). EMT and Notch-related genes are shown. (E) Representative western blot showing GSI RO treatment followed by NCST increase. Notch4 cleavage is increased (50 KDa) or unaffected. Total protein was normalised to Actin (N = 3 independent experiment, bars show SD). [file bcr3675-S4.pdf]

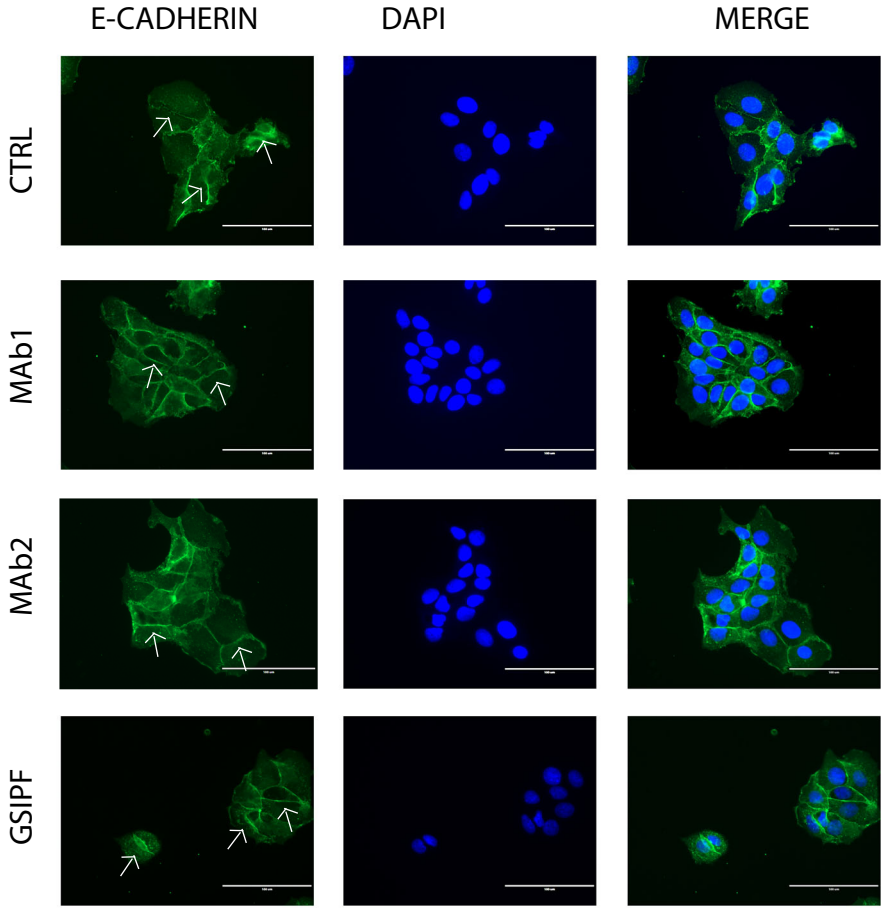

Supplement: Additional file 5: Figure S4 — Representative images showing E-cadherin localization in tamoxifen-resistant (TAM-R) cells treated with control immunoglobulin G (IgG), monoclonal antibody 1 (mAb1), monoclonal antibody 2 (mAb2) and gamma secretase inhibitor Pfizer (GSIPF). [file bcr3675-S5.pdf]

**S1A**

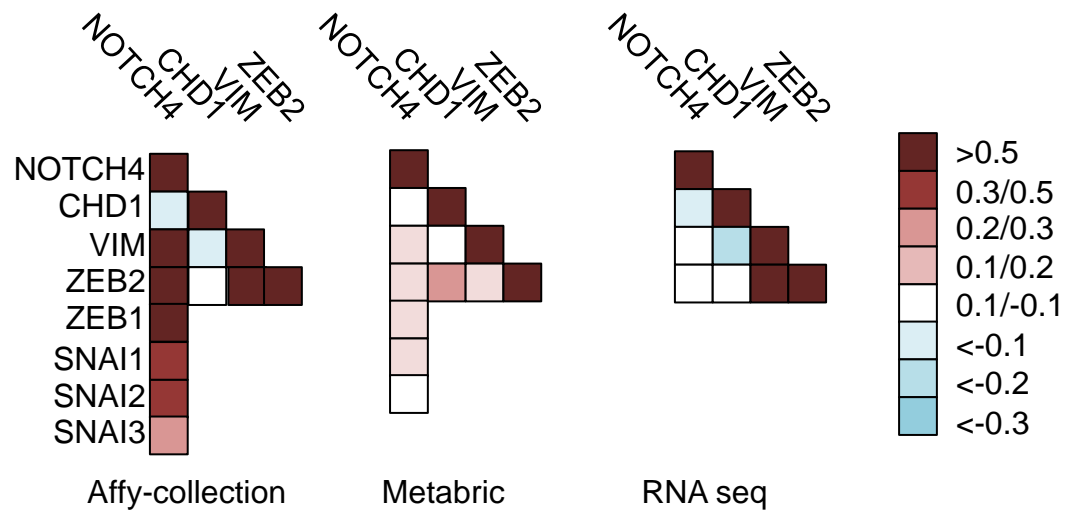

**S1B**

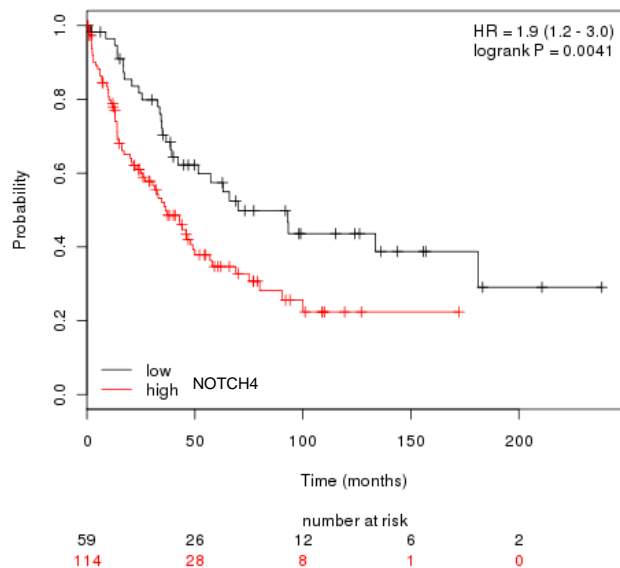

Supplement: Additional file 6: Figure S5 — (A) Pearson correlation coefficient between RNA-seq data shows that high expression of Notch4 correlate with high expression of VIM, ZEB1/2 and SNAI1/2/3 while correlating with low expression of E-cadherin (CHD1). (B) Kaplan-Meier model comparing post-progression survival in estrogen receptor alpha (ERα)-positive breast cancer patients showing Notch4 expression. [file bcr3675-S6.pdf]
